# Better to Have Loved and Lost? Human Avoidant Attachment Style Towards Dogs Predicts Group Membership as ‘Forever Owner’ or ‘Foster Carer’

**DOI:** 10.3390/ani10091679

**Published:** 2020-09-17

**Authors:** Jannine M. Lockyer, Jessica L. Oliva

**Affiliations:** School of Psychological Sciences, Faculty of Medicine, Nursing and Health Sciences, Monash University, 3800 Melbourne, Australia; janninelockyer@gmail.com

**Keywords:** attachment, assistance, dog, puppy, SED

## Abstract

**Simple Summary:**

Early attachments to human carers may have long lasting impacts on a seeing eye dog’s working life. Using a self-report questionnaire, we found that puppy carers demonstrated more avoidant styles of attachment towards the dogs in their care as compared to dog owners. Carers also predominately appeared to be driven to the puppy caring role by more pro-social motivations that do not relate to the dog itself. Carers’ reluctance to form close relationships with the puppies in their care may impact subsequent bond formation with other humans, as well as their working performance and wellbeing.

**Abstract:**

Important physiological, performance, and relationship differences have been reported between companion and working dogs. This study aimed to investigate how human attachment styles manifest towards dogs, depending on the dog’s role. Seeing Eye Dog (SED) carer (*n* = 25) and Companion Dog Owner (CDO) (*n* = 78) avoidant and anxious attachment scores towards the dog in their care were compared. Feelings and motivations about being a SED carer or CDO were also investigated qualitatively. Significant differences were observed between pet avoidance, with avoidance scores significantly predicting SED carer group membership. Qualitative insights revealed more human prosocial motivations for becoming a SED carer, and more dog-related motivations for becoming a CDO, with CDOs more likely to consider their dog a ‘significant other’. This study corroborates findings supporting differences in human–dog relationships for working versus companion dogs. The potential impacts of human–dog attachment should be considered for SED success.

## 1. Introduction

Seeing Eye Dogs (SED) perform a vital function in the community, facilitating independence and enhancing the quality of life for those who are vision impaired [1]. Based on population growth estimations, Vision Australia forecast increased demand for SED services, with 564,000 people in Australia estimated to be classified as blind or low vision by 2030 [2], a 47% increase from 2016. In 2009, the cost to the economy in lost earnings of blind or vision-impaired people was estimated at $2.3 billion [3]. Furthermore, lost earnings for carers was estimated at $251 million [3]. The average cost of care and training for a SED is estimated to be $50,000 [2]. Furthermore, graduation rates for guide dogs are at approximately 50% [4]. Given projections of increased demand for support services [5], together with low graduation rates of SED [4], initiatives that may support SED puppy progression rates are worthwhile.

### 1.1. Influences on Puppy Development

Interviews with 63 vision impaired individuals who currently have SED assistants indicated highly desirable SED behaviours included attentiveness to command or task, confident decision making, and calmness [6]. In relation to SED puppy training, leash-pulling, reduced responsiveness to control or trainability, and high scores on temperament measures associated with anxiety, distractibility, excitability, and body sensitivity have all been demonstrated to reduce the possibility of graduation [7,8,9]. Similarly, enhanced reactivity to unfamiliar environments, like a dark room or noisy metal stairs, has been shown to be predictive of other fearful behaviours in the home, like cowering and reactions to other dogs [10]. Harvey et al. [11] revealed that puppies socialised with other dogs exhibit lower separation-related behaviour and higher trainability, and this has been associated with SED success in Australia [7]. As SED carers usually care for SED puppies from 8 weeks of age until the dogs are approximately 12 and 15 months old [2], social interactions in carer home environments likely influence SED puppy behavioural development. Mai et al. [12] have highlighted several important self-report human factors that might influence SED puppy success, including unrealistic expectations, level of competency, perseverance, and passion. However, the carer–puppy relationship warrants further attention, as limited research has been conducted into how carers may influence the development of temperament and the impact on socialisation of puppies during this period.

### 1.2. Human–Dog Attachment

The human–dog dyad shares similar characteristics to the human infant/caregiver dynamic which may therefore have implications for dog behaviour [13]. According to Bowlby’s [14] theory of attachment, (later expanded upon by Ainsworth [15]), infants develop a ‘style’ of attachment in response to the nature of responding of their primary caregiver. These styles can be broadly classified as ‘secure’ or ‘insecure’. Insecure attachment styles can then be further characterised as either ‘anxious’ or ‘avoidant’. Anxious and avoidant behaviours initially emerge as a protective and adaptive response to inconsistent caregivers’ behaviours [16]. However, it has been postulated that the internal working ‘model’ of attachment developed in infancy is carried forward into adulthood, for a review see [17], and long-term indiscriminate application of anxious or avoidant orientated working models may not be self-serving [18].

The internal working model of attachment expressed in adulthood is known as one’s adult attachment style (AAS) [19], and can be applied to romantic attachments [20], as well as attachments to pets [21,22]. While early interactions are most influential to one’s AAS, these ‘working models’ are continuously being updated based on new information and new relationships [17]. Furthermore, one′s expectations about interactions with others can differ depending on the relationship in question, and past experiences [17]. Research comparing AAS towards humans and pets indicates similarities between attachment styles, but also some differences. For example, Zilcha-Mano et al. [22,23] found anxious pet attachment scores had a moderate and positive correlation with measures of human attachment anxiety. However, the same researchers have shown inconsistent associations between anxious pet attachment and human avoidance. These inconsistencies might be explained by their sample of mixed pet owners. Brown and Symons [24] also found moderate positive correlations between measures of human attachment and pet (cat and dog) attachment, however, results differed whereby avoidant pet attachment related to both human avoidant and anxious AAS [24]. Although all studies used the Pet Attachment Questionnaire (PAQ) [22] to measure AAS towards pets, different versions of the Experience in Close Relationships Scale (ECR) were employed to measure AAS towards other humans. Furthermore, differences in findings may also be due to the retrospective nature of the Brown and Symons [24] study which required participants to complete the PAQ after their pet had passed away, which may have complicated participant’s relational perspectives. Nevertheless, it seems owners’ AAS does manifest in attachment towards pets, however, there may be differences in how pet anxious and avoidant attachment dimensions are expressed.

Characteristics and measures of human infant attachment are also evident in dogs towards their owners, including: proximity seeking, separation anxiety, utilising safe haven and safe base effect when distressed, responses to stranger or challenging situation, vocalising or unsettled behaviours, and play when owner is present compared to absent [25,26,27,28]. Given that dogs reach social maturity between the ages of 12 to 36 months [29] and that puppy carers are in charge of SED puppies between the ages of 2 to 15 months it is probable they fulfil a similar role to Companion Dog Owners (CDOs) in the puppy’s life, being their most significant person and may act as an attachment figure. It follows that SED carers are probably influential in the maturation or development of the puppy during this period. Hence, it is possible puppy carer AAS influences the way the carer interacts with the dog and in turn, the dog’s temperament, or the behavioural development.

Indeed, anxious and avoidant pet attachment scores have been associated with negative expectations of pet behaviours, and the pet owner’s trust, closeness, and dependability of the pet [22]. Furthermore, AAS of owners has been associated with dogs’ performance on cognitive tasks [30,31] and avoidant AAS of owners has been associated with separation anxiety disorder in dogs [32]. Oliva et al. revealed that the AAS of French puppy carers were on average more avoidant than CDOs [33], and while the dogs raised by these more ‘avoidant’ carers were more likely to participate in a cognitive task, they were no more likely than pet dogs to be accurate. They also demonstrated higher levels of prolactin in their blood [33], which has been associated with emotional disorders in anxious dogs [34]. Thus, there is compelling evidence for an association between owner attachment style and dog behaviours. Reasons for significant differences in avoidant attachment style of CDOs compared to SED carers is unclear, however this finding warrants further investigation into how this might be impacting dogs destined for working roles.

This study aims to investigate AAS in relation to the role of the dog, being SED or companion dog, by comparing participants on AAS towards humans and AAS towards dogs. We hypothesise that people with more avoidant working models of attachment will be more psychologically equipped to deal with the inevitable loss of a SED dog after 12–15 months of caring for it and will therefore be more attracted to this role. Hence, we expect to see greater levels of avoidant attachment in this cohort, both towards their dogs as measured by the PAQ, and towards other humans as measured by the ECR-R, as compared to CDOs. Conversely, we hypothesise that people with more anxious working models of attachment will be attracted to the idea of a ‘forever’ dog, and hence we expect greater levels of anxious attachment will be demonstrated in the cohort of CDOs, both towards their dogs, and other humans. The capacity of subscale scores to predict type of carer (SED or CDO) will also be investigated where it is hypothesised avoidant attachment will predict SED carers and anxious attachment will predict CDOs. Finally, the association between subscale scores and graduation outcomes will be investigated.

## 2. Materials and Methods

### 2.1. Participants

One hundred and five participants aged 26–75+ years completed the study. Group one was comprised of SED carers (*n* = 27; *men* = 5, *women* = 22) who responded to an email invitation sent directly from Vision Australia. Group two comprised CDOs who responded to communications targeting responsible dog owners (*n* = 78; *men* = 14, *women* = 63, *non-specified* = 1) disseminated via social media forums, snowballing, and the researchers’ personal networks. Fifty two percent (14) of SED carers were under the age of 55 and 48% (13) were 56 or older. For CDO, 77% (60) were under the age of 55 and 23% (18) were 56 or older.

No inducements were offered or given to participants. Criteria for inclusion in the study included the age of the dog (minimum 12 months), the time the dog has been in the home with the SED carer or CDO (minimum 10 months), and the recency of contact between SED carer and SED dog or CDO and dog (maximum four week separation at time of survey). For 26% of SED carers this was their first experience being a carer. Participants were excluded if they self-reported as having been convicted for animal cruelty or irresponsible sourcing of a pet (for example from a disreputable source or puppy farm). For further information please refer to Table 1 which shows the demographic characteristics of participants in the final sample. This study received ethical approval from Monash University Human Research Ethics Committee on 8 August 2019 (project ID: 20871).

### 2.2. Measures

The online survey was hosted by Qualtrics and included demographic questions, the PAQ, two short-answer questions, and the ECR-R. Demographics questions included dog age, time in home, pet ownership history, parenting history and for SED carers only, SED carer history.

The PAQ [22] is a 26-item self-report questionnaire measuring adult attachment orientations of people towards their pets. Respondents were asked to reflect on their feelings about and experience of, their current pet relationship. For the purposes of the current study, the word ‘pet’ was replaced with the words ‘pet dog’ or ‘SED puppy’, as appropriate. Individuals responded to each statement by indicating their level of agreement on a seven-point Likert scale (1 = Strongly disagree, 7 = Strongly agree). Thirteen items on the scale relate to avoidant attachment towards pets (e.g., “I’m not very attached to my pet” or “I have no problem parting with my pet for a long duration”). The remaining items investigated anxious attachment towards pet (e.g., “I am worried about being left alone without my pet” or “I need shows of affection from my pet to feel there is someone who accepts me as I am”). Participants’ individual scores on the attachment dimensions of avoidant and anxious were calculated after reversed keyed items were reverse scored [22], and by dividing these totals by 13. Higher scores relate to higher anxiety or avoidance attachment orientation. Both subscales have demonstrated good test re-test reliability (r = 0.75 for anxiety and 0.80 for avoidance) and good internal consistency (α = 0.86 for anxiety and 0.87 for avoidance) [22]. The PAQ has been reported to have good convergent and discriminant validity across several studies [22]. Cronbach alphas indicated good reliability for PAQ avoidant (α = 0.81) and anxiety (α = 0.81) items in the current study.

The ECR-R [35] is a 36-item self-report questionnaire commonly used to measure adult attachment orientations towards humans in close relationships on the sub-scales of avoidant and anxious attachment. In this study, respondents were asked to reflect on how they felt in close relationships and their general experience of intimate relationships, regardless of relationship status. In responding to statements, participants indicated their level of agreement on a seven-point Likert scale (1 = Strongly agree, 7 = Strongly disagree). Anxious attachment orientation was gauged over 18 items (e.g., “I worry a lot about my relationships” or “My desire to be very close sometimes scares people away”). An additional 18 items were used to investigate avoidant attachment (e.g., “I rarely worry about my partner leaving me” or “I find it difficult to allow myself to depend on romantic partner”). An individual’s scores on each subscale were averaged after reversed keyed items were reversed scored [35]. Higher scores relate to higher anxiety or avoidance attachment orientation. A meta-analysis of 242 studies indicated the ECR-R to be a reliable measure of adult attachment orientation dimensions [36]. Sibley and Liu [37] found subscales of anxious and avoidant attachment were stable and internal consistency was high for both subscales, with Cronbach alphas of 0.95 and 0.93 for anxious attachment and of 0.93 and 0.91 for avoidant attachment. Factor analysis has confirmed the two attachment dimensions [37]. Cronbach alphas in the current study were excellent for ECR-R avoidant (α = 0.95) and anxiety (α = 0.95) subscales.

#### Qualitative Questions

Two qualitative questions were included to complement the PAQ and ECR-R measures. Firstly, participants were asked to describe in a few sentences why they decided to become a SED carer or CDO. Secondly, SED carer and CDOs were asked to describe their feelings towards SED or CD in comparison to significant others in their life.

### 2.3. Procedure

During a period of six weeks of data collection, interested participants used their own personal devices and clicked on a hyperlink in the SED invitation email or CDO advertising to access the survey anonymously, on the secure web-based platform Qualtrics. The group designated hyperlinks connected participants to the SED survey or the CDO survey. No identifying information of participants was collected. Consent was implied when participants completed the survey. Two animal welfare exclusionary questions were presented to CDOs only as SED carers had been vetted in SED processes. CDO respondents who answered ‘yes’ to a conviction of animal cruelty or to sourcing a dog irresponsibly, for example from a disreputable source or puppy farm, were transferred to the end of the survey and thanked for their participation. Next, participants completed demographic questions relating to the dog in their care, including dog age, breed, time in home, also pet ownership history, parenting history and for SED carers only, SED carer history. The demographic questions were followed by the multiple-choice PAQ and two short answer questions. Lastly, participants completed the ECR-R multiple choice questionnaire and then were thanked for their time.

## 3. Results

The raw data was exported from Qualtrics to SPSS v26. Please see Table 1 for demographic characteristics of the sample.

### 3.1. Men Compared to Women

A preliminary analysis to investigate potential group differences between average scores for men and women was undertaken as research indicates individual attachment differences are often multidimensional [22]. One participant who did not specify their gender was not included in the analysis. For males, a visual inspection of histograms and Q-Q plots for subscale variables of ECR-R and PAQ indicated normal distributions for all subscales. For females, histograms and Q-Q Plots indicate some minor deviation from normal distribution where scores are weighted towards a positive skew (i.e., lower scores) for the subscales of ECR-R and PAQ Anxious and Avoidant, indicating more secure attachment styles. Extreme outliers were checked using z scores ≥ ±3.29 and one was found in the female PAQ Avoidant scale data. To ensure this data point was not exerting undue influence on the model, its Cook’s distance was checked and found to be <1 at 0.17 and therefore retained as it was not exerting undue influence. Levene’s test of equality of variances indicating variances were similar for all subscale variables for SED and CDO groups (*p* > 0.05).

As per Table 2, independent samples *t*-tests with bootstrapping applied to account for the slight deviations from normality in the data revealed no differences between males and females on any of the subscales and so aggregate data from males and females combined was used for subsequent analyses involving these scales.

### 3.2. Group Comparison of SED Carers and CDOs

Visual inspection of histograms and Q-Q plots indicated a relatively normal distribution of subscale scores for the SED and CDO populations with exception of SED ECR-R Anxious, and CDO PAQ Avoidant scores which were positively skewed to lower scores. Once again, Cook’s distance was checked for the outlier > ±3.29 in the PAQ Avoidant dataset and was not found to be exerting undue influence on the model with a Cook’s distance of 0.07, and was therefore retained. Levene’s test of equality of variances indicating variances were similar for all subscale variables for SED and CDO groups (*p* > 0.05).

Independent samples *t*-tests with bootstrapping applied to account for the slight deviations from normality in the data were performed to investigate average differences between groups on the subscales of ECR-R Avoidant, ECR-R Anxious, PAQ Anxious or PAQ Avoidant. As per Table 3, results indicate SED carers on average were higher on scores of PAQ subscale avoidant compared to CDO. This effect is large.

Pearson’s correlations were performed to investigate the association between the subscales of ECR-R Avoidant, ECR-R Anxious, PAQ Anxious, or PAQ Avoidant. Table 4 indicates significant correlations between ECR-R Anxious and ECR-R Avoidant subscales, the ECR-R Anxious and PAQ Anxious subscales, and the ECR-R Avoidant and PAQ Anxious subscales.

A forced entry binary logistic regression analysis was performed to assess the predictive ability of the subscales for group membership, with SED carer as the target category. One overly influential case in the CDO group was identified with a Cooks distance of 1.63 and was therefore deleted from the logisitic regression analysis. The Omnibus Tests of Model Coefficients was statistically significant $x^{2}$ (*df* = 4, *N* = 104) = 36.74, *p* < 0.001. No issues with multicollinearity were evident and a logistic regression was performed to assess the linearity of the logit. Linearity assumptions were met where interaction terms for ECR-R Anxious, ECR-R Avoidant, PAQ Anxious, and PAQ Avoidant were non-significant (*p* > 0.05). All assumptions were satisfied for a logistic regression analysis.

In the absence of any predictors, 74% of cases could be accurately classified. This increased to 81.7% with predictors in the model, whereby prediction sensitivity was 52% for correct classification of SED carer membership and 96% specificity in predicting CDO membership. As per Table 5 below, the initial bivariate models demonstrate that the PAQ avoidant subscale positively and significantly predicted SED carer group membership, whereas PAQ anxious and ECR-R Avoidant demonstrated a negative, non-significant relationship with it. Bivariate analysis indicates ECR-R anxious appears to be a positive predictor, however this relationship changes to negative in the logistic regression. This shift in direction of effects could be suggestive of a suppression effect.

Hosmer and Lemeshow test $x^{2}$ (8, *N* = 104) = 7.88, *p* = 0.445, indicates good model fit, where there is no significant mismatch between actual and predicted group membership. The model explained between 30% (Cox and Snell $R^{2})$ and 44% (Nagelkerke $R^{2})\;$ of the variance for SED carer group membership. As per Table 6, the logistic regression model was statistically significant, $x^{2}\;$(1) = 22.04, *p* < 0.00, 95% CI [4.63, 41.74]. Of the four predictors, only the PAQ avoidant subscale was a significant predictor of target group (SED carer) membership, whereby there is a 1391% increase in the odds of being a SED carer, with a 1 unit increase in PAQ avoidant scale. That is, as avoidance scores increase, so do the odds of being a SED carer.

### 3.3. SED Carer Attachment and SED Puppy Success Rates

Finally, graduation rates of SED were followed up with Vision Australia and associations between graduating (*n* = 9) and non-graduating (*n* = 18) dogs were correlated with PAQ Anxious and Avoidant subscales scores using a point-biserial correlation. Preliminary analyses showed there were (a) no outliers as assessed by z-scores >3.29; (b) avoidant and anxious attachment scores were normally distributed in the SED carer population as assessed by Shapiro-Wilk’s test (*p* > 0.05); and (c) there was homogeneity of variances as assessed by Levene’s test for equity of variances. No significant correlations were found between puppy graduation and SED carer PAQ Avoidant scores (*r_pb_*(27) = 0.12, *p* = 0.56), nor between puppy graduation and PAQ Anxious scores (*r_pb_*(27) = 0.20, *p* = 0.31), however, post-hoc power analyses revealed that there was insufficient power to detect any significant differences (ß − 1 = 0.45 and ß − 1 = 0.70, respectively).

#### Qualitative Analysis

Conventional content analysis was applied to the individual responses to the open-ended questions. It was decided prior to analysis that content would be referenced only once. Two independent raters reviewed the first five responses for each question, independently allocating content to labels or ‘themes’. The continued refinement, sub-categorisation, and synthesis of these themes was progressive throughout the evaluation of discrete blocks of 10 responses per question, after which they would meet and resolve any disagreements through discussion. This process continued until inter-rater reliability of ≥0.8 was achieved for each question. Themes with minimum 5% participant endorsement were retained.

As shown in Table 7, motivations for becoming a SED carer were largely pro-social or self-related, whereas motivations for becoming a CDO were predominantly dog related positive affect based. Themes relating to motivations for SED carers and CDO individually are presented in Table 8 and Table 9, respectively.

As shown in Table 10, feelings towards the dog in their care differed between SED carers and CDOs whereby SED carers predominately felt their dog was not comparable to significant others in their life but role related, and CDOs predominately felt their dog was comparable to significant others in their life or more.

Themes relating to SED carers and CDO individual feelings towards their dogs are presented in Table 11 and Table 12, respectively. It is relevant to note that, in response to this question, a large number of participants provided responses reflecting positive emotions, experiences or interactions with their dogs (*n* = 29 (37%) for CDOs and *n* = 4 (15%) for SED carers), however, given this ‘theme’ does not relate to the question, these responses were omitted from the analysis.

## 4. Discussion

The study aimed to replicate findings by Oliva et al. [33] that demonstrated that puppy carers were more avoidant towards the dogs in their care, as compared to CDOs. The current study also aimed to extend on these findings by investigating whether human attachment styles towards other humans was associated with human attachment styles towards dogs, and whether these attachment styles could predict group membership as either a CDO or SED carer. Furthermore, the study investigated whether SED directed attachment was associated with SED graduation rates. In addition, two qualitative questions investigated the original motivations of SED carers and CDOs in caring for their dog, as well as their current feelings towards their dog as compared to significant others. Our hypotheses were partially supported, in that SED carers demonstrated significantly more avoidant attachment styles towards the dogs in their care, in line with Oliva et al. [33], but not towards other humans. No significant differences were observed between SED carers and CDOs in regards to anxious attachment towards the dogs in the care, nor to other humans. Group membership as either a SED carer or CDO was able to be determined based on PAQ avoidant scores. The association between subscales scores and graduation rates was not able to be determined due to lack of power likely resulting from the small sample size of SED carers.

This study found significant moderate and positive correlations between ECR-R subscales of Anxious and Avoidant, ECR-R Anxious and PAQ Anxious, and ECR-R Avoidant and PAQ Anxious. No associations were found between PAQ Avoidant and any of the other subscales. The association between anxious attachment to people and anxious attachment to pets is consistent with previous literature [22,23,24], however the null association between anxious attachment to people and avoidant attachment towards pets is largely inconsistent. However, these prior associations were only small, and observed using different variations of the human attachment scale (the ECR-RS and the ECR, respectively), in a sample of mixed pet owners. The association between avoidant attachment towards humans and anxious attachment towards pets is in line with findings from Zilcha-Mano et al. [22], but contrary to Brown and Symons [24] who found that avoidant attachment towards people was associated with avoidant attachment towards pets, as well as Zilcha-Mano et al. [23] who found that avoidant attachment towards people was not associated with either subscale of the PAQ in their study 1 and associated with pet avoidance in their study 2. Notably, average human avoidant and anxious scores were higher in Zilcha-Mano et al.’s [23] study in comparison to current findings, and the sample consisted of both dog and cat owners. These inconsistencies might also be explained by the use of different versions of the questionnaire across studies, as well as the retrospective design used by Brown and Symons [24] who asked people to reflect on their attachment towards a pet that had passed away. In line with Zilcha-Mano et al. [22,23], the current findings support an orthogonal relationship between avoidant and anxious attachment to pets, however, anxious and avoidant attachment to humans demonstrates some overlap, as was also observed by Brown and Symons. That avoidantly oriented people can form more anxiously oriented attachments to their dogs demonstrates the important role a dog may play in their owners’ lives, enabling them the opportunity for closeness and vulnerability that they are generally not afforded, which may in turn modify their working model of attachment in a way they are not able to achieve with other humans.

It is unclear whether an avoidantly oriented person becomes more anxiously oriented to their dog through the enduring owner-pet relationship, or whether their avoidant nature is associated with anxious attachment to their dog from the beginning of their relationship. The latter would be in contrast to our theory that avoidantly attached people are more psychologically equipped to deal with the inevitable loss of a dog, and indeed, SED carers who are faced with this loss approximately 1 year into their relationship, do show significantly more pet avoidance than CDOs. This would suggest more anxious attachment towards pet dogs may develop over time in people with avoidant attachment orientations. Hence, significant differences between SED carers and CDO on pet avoidant attachment may also be due to limited bonding time. Reevy and Delgado [38] used the PAQ on a large sample and reported that lower pet avoidant attachment scores were related to longer time being a CDO and being female. In the current study, using an Australian-based sample, the CDO average time for dogs in home (5.4 years) was approximately five times greater than the SED group (1 year) time of SED in the home. In addition, 81% of the sample were female, which was proportionally the same for both groups. Therefore, the time in home differences observed between groups in the current study could explain some of the difference between group mean PAQ avoidant scores.

Qualitative findings corroborate the idea that SED carers and CDOs possess divergent motivations in caring for their dogs (refer to Table 7, Table 8 and Table 9). For example, the most prominent motivations for becoming a CDO were dog-related (64%). In comparison, SED carer motivations were prosocial (49.1%), relating to others and wanting to contribute to the community. In describing feelings towards the dog in their care compared to significant others (Table 10, Table 11 and Table 12), CDOs more frequently endorsed feelings ‘comparable to significant others or more’ (58.5%), whilst SED carers more commonly endorsed feelings ‘not comparable to significant others’, ‘role related’ or ‘comparable but less’ (69.1%). In describing their motivations and feelings, CDO responses were focused more on positive affect towards or about the dog, whereas SED carers related more to helping behaviours, the role of being a carer and indicated less emotional connection to the dog in their care compared to CDOs. Carer perceptions of the role they play in a SED puppies’ life may cause them to avoid or limit attachment. While understandable, the potential effects of an avoidant human attachment style on dog development warrants further consideration.

A variety of human characteristics and attitudes have been reviewed and said to influence the human–dog relationship [13]. These include affiliative, attentive, or dominionistic behaviour and positive, empathetic, or negative attitudes towards the dog. It appears consistent affiliative and positive interactions between human and dog promote a secure bond where both parties experience emotional and physiological benefits, enhancing the wellbeing of each [13]. Hence, it is also probable that an insecure attachment has an effect on dog wellbeing. Later in life, avoidant AAS are sometimes reflected in relational behaviour with individuals demonstrating less intimacy seeking and suppression of emotions [18]. Relative to dogs, tentative research using global measures indicated CDOs high on human avoidant attachment style measures reported less pet-related concern, caregiving, and attentiveness towards their dogs [39]. Furthermore, CDO human avoidant attachment has been associated with separation anxiety in dogs [32]. Potential physiological indicators of dog anxiety [34] are also shown to be higher in SED puppies compared to CDOs, whose carers demonstrate significantly more avoidant attachment towards them [33]. The results may imply human attachment style affects the human–dog dyad. While no differences in human directed AAS were found in the current study, SED carers showed a trend for lower ECR-R anxious scores as compared to CDOs (refer to Table 3). However, the bivariate relationship between ECR-R anxious scores and SED carer membership suggested the opposite i.e., an increase in odds of being a SED carer with increasing ECR-R anxious scores (refer to Table 5). This may suggest a suppression effect and warrants further investigation in future research, in a larger sample of SED carers.

The effects of attachment style on the human–dog dyad also poses questions about resulting human perspectives on dog behaviour. High pet avoidant attachment has been associated with negative expectations of dog behaviour [22]. Payne et al.’s [13] review suggests negative attitudes towards a dog probably lead to poor signalling to the dog and poor human–dog communication creating the potential for humans to underestimate a dog’s cognitive ability. Indeed, Oliva et al. [31] demonstrated that anxious attachment towards dogs was negatively associated with pet dog’s ability to perform a cognitive task using pointing cues. Conversely, avoidant attachment towards dogs was associated with owners’ perceptions that their dog can pick up on their emotions, which was positively associated with performance on the same task using gazing cues. This indicates that there might be differences in the way avoidant versus anxiously oriented carers communicate with their SED dogs, and this in turn might impact the dog’s ability to perform. Certainly, if dogs look to the eye region of more avoidant carers for cues about how to complete a task, this could be problematic down the line when they are placed with a visually impaired client.

## 5. Limitations and Future Directions

A strength of this study is the exclusive focus on SED carers. Potentially, carers or handlers of border control dogs or other types of assistance dogs could be differentially motivated, where carer instruction and dog purpose differ. However, we acknowledge that there may have been overlap between SED carer and CDO group membership with approximately 50% of SED carers were also pet dog owners and 66% had pets other than dogs. Another strength was that a similar percentage of participants in both groups were parents, as this has been shown to be related to pet attachment style, with parents more avoidant than non-parents [31]. Unfortunately we were unable to match for dog age across the two groups, as most of the pet dogs were past the puppy stage, which is recognized as a challenging life stage for carers to manage [40]. Hence, future studies could control for this by including CDOs with dogs of similar ages to the SED population. It would also be interesting for future studies to consider attachment from the dog’s perspective towards their owners/carers and how this might be associated with the human attachment style directed to them. This study might also serve as an interesting model for future studies to investigate bidirectional parental–child attachment in fostered versus adopted (i.e., “forever”) children.

The study was also limited by the small pool of potential SED carer participants (i.e., those who received Vision Australia’s email; *n* = 69) which yielded a small SED sample size (*n* = 27) compared to CDO group (*n* = 78). The response rate of approximately (39%) was lower than SED Australia and researchers’ expectations. Contacts from SED Australia indicated potential reasons for this, drawn from their understanding of SED carers, include the fact that carers are often late adopters and limited users of technology which was required for participation in the online survey, carer concerns about being deemed ineligible to remain puppy carers, and some carers thinking they were ineligible. While the study was sufficiently sensitive to find a significant result and a substantial effect size for differences between SED carers and CDO on pet attachment, it was insufficiently powered to detect associations between SED carer pet attachment and SED puppy graduation rates, hence, future studies should aim to replicate these analyses in a larger sample.

The increasing public consciousness around working dog welfare [41] could offer support for initiatives that may improve it. For example, SED organisations perceived as leaders in dog welfare, may seek opportunities for additional research around improvements to SED carer behaviours towards their trainee dogs. This may strengthen their position as a knowledge base for promoting and disseminating education that enhances the welfare of dogs. Additionally, increasing the awareness of SED carer and CDO attachment styles and possible related care-giving patterns or behaviours toward dogs, may offer opportunities to enhance interactions with the dogs in their care. The benefits of greater understanding and increased awareness, could provide a level of reciprocity where trainers and owners work together to reduce stress on dogs, resulting in a more responsive dog with greater wellbeing.

In a broader sense, further research on the impacts of human attachment styles on the human–dog dyad, could offer insight for those supporting the development and sustenance of positive human–dog bonding. Knowledge of the effects of attachment styles could inform human–dog matching for shelter or assistance dog organisations. As noted, greater understanding could lead to improved care-taking behaviours, interpretations of dog response, and training outcomes. Additionally, evidence of the effects of human attachment on dogs could prove useful in a variety of settings. For example, veterinarians, dog or pet behavioural advisors, kennel operators, trainers, animal shelters, assistance dog organisations, and carers. The potential benefits for stakeholders, and the enhanced wellbeing for humans and dogs, are valid motivators for further research.

## 6. Conclusions

This research adds to existing knowledge that attachment styles towards dogs manifests differently, depending on the role of the dog in the carers life. Specifically, SED carers are more avoidant in their attachment orientations as compared to CDOs, however this was only evident in their human–dog relationships, and not their human–human ones. This was corroborated by qualitative findings suggesting that motivations to become a SED carer are removed from the human–dog relationship and more related to helping other people in the community, which is in stark contrast to CDOs, whose motivations were predominately dog-related. Current literature indicates human–dog attachment may have psychophysiological impacts on dogs [30,31,33]. Therefore, potential effects of human attachment on the human–dog dyad should be considered and highlighted to carers if SED are to have the best opportunity for success. Furthermore, investigations into human attachment styles and associated behaviours in relation to dog behaviour are needed to illuminate relational dynamics and discover the causes and effects of human psychological characteristics on dog behavioural responses.

## Figures and Tables

**Table 1:** Parental and Pet-Keeping history of Participants and Demographics of Their Study Dog.

Base Line Characteristic	SED Carers (*n* = 27)		Companion Dog Owners (*n* = 78)	
	*n*	(%)	*n*	(%)
Average number of participants identifying as parents	20	74	51	65
Average number of parented children	2.4	-	2	-
Average number of children in home	2.2	-	2.9	-
Number of participants with a second or more dog now or previously	14	52	59	76
Average number of previous or current companion dogs excluding the dog of focus for survey	2.5	-	2.7	-
Number of participants with other pets	18	66	52	67
Average number of other pets	3.4	-	3.8	-
Dog Sex				
Male	13	48	32	41
Female	14	52	46	59
De-sexed				0
Yes	22	81	72	92
No	5	19	6	8
Average age of dog (years)	1.4	-	7.5	-
Average time of dog in home (years)	1	-	5.4	-

**Table 2:** Results of Comparisons of Gender on Subscale Variables of Attachment.

Subscales	Males		Females		*t*(101)	*p*	*Cohen d*
	*M*	*SD*	*M*	*SD*			
ECR-R Anxious	2.69	0.96	2.44	1.19	0.84	0.402	0.23
ECR-R Avoidant	2.76	1.15	2.47	1.08	1.05	0.298	0.26
PAQ Anxious	2.34	0.58	2.19	0.69	0.90	0.372	0.24
PAQ Avoidant	1.91	0.67	1.65	0.66	1.54	0.126	0.39

Mean values for each of the subscale parameters are shown for males (*n* = 19) and females (*n* = 85), together with *t* tests (assuming equal variance) comparing the variables for the two groups.

**Table 3:** Results of Comparisons of Groups on Subscale Variables of Attachment.

Subscales	SED		CDO		*t*(101)	*p*	*Cohen d*
	*M*	*SD*	*M*	*SD*			
ECR-R Anxious	2.28	1.14	2.56	1.14	−1.11	0.272	0.25
ECR-R Avoidant	2.43	0.982	2.55	1.13	−0.50	0.619	0.11
PAQ Anxious	2.15	0.656	2.23	0.679	−0.55	0.584	0.12
PAQ Avoidant	2.26	0.613	1.51	0.574	5.74	<0.001	1.26

Mean values for each of the subscale parameters are shown for Seeing Eye Dog (SED) (*n* = 27) and Companion Dog Owner (CDO) (*n* = 78), together with *t* tests (assuming equal variance) comparing the variables for the two groups.

**Table 4:** Correlations for Study Variables.

Variable	1	2	3	4
1. ECR-R Anxious	-			
2. ECR-R Avoidant	0.58 **	-		
3. PAQ Anxious	0.54 **	0.40 **	-	
4. PAQ Avoidant	0.05	0.12	0.03	-

** *p* < 0.001.

**Table 5:** Bivariate Relationships between Subscales and SED carer Group Membership.

Variable	*Exp (b)*	*p*
ECR-R Anxious	1.08	0.299
ECR-R Avoidant	0.23	0.630
PAQ Anxious	0.31	0.577
PAQ Avoidant	33.27	<0.001

**Table 6:** Results of Binary Logistic Regression predicting SED carer Group Membership.

Variable	B	SE	Wald	*p*	*Exp (b)*	95% CI
ECR-R Anxious	−0.17	0.39	0.18	0.665	0.84	[0.39, 1.83]
ECR-R Avoidant	−0.26	0.39	0.43	0.511	0.78	[0.36, 1.66]
PAQ Anxious	−0.30	0.55	0.29	0.588	0.74	[0.26, 2.16]
PAQ Avoidant	2.63	0.56	22.04	0.001	13.91	[4.63, 41.74]

$R^{2}$ = 0.29 (Cox and Snell) 0.44 (Nagelkerke). PAQ subscale avoidant was a significant predictor in increasing the odds of being in the target group of SED carer. *N* = 104.

**Table 7:** Meta Theme Endorsement for Motivations for Becoming a SED Carer Versus CDO.

Meta-Theme	SED Carer		CDO	
	Number of Endorsements	(%)	Number of Endorsements	(%)
Prosocial based	30	49.1	33	25.8
Self-related	22	36.1	12	9.4
Dog related positive affect-based	9	14.8	83	64.8

Percentages were calculated by dividing the number of endorsements for each individual meta-theme by the total number of endorsements made across all meta-themes. Individual participants were only able to endorse a theme once but were able to endorse more than one theme within the same meta-theme.

**Table 8:** SED motivations for becoming a SED carer (*n* = 27).

Meta-Theme	Theme	Sub-Theme	Example Quote	Frequency, *n* (%)
Prosocial based motivation	Give back to community/volunteer		“Both my husband and I feel it is about giving something back to the community.”	23 (85.2%)
	Persuaded/suggested by others		“Our neighbours were SED carers, and persuaded us to give it a try.”	4 (14.8%)
	For own child/family		“…need to get child more confident with dogs, understanding the degree of care needed…”	3 (11.1%)
Self-related motivation	Unwilling to commit to a pet dog	Too old	“We have always had dogs in the family but didn′t want to commit to the long-term responsibility of owning another dog of our own at our ages 70 plus.”	13 (48.2%)
		Unwanted responsibility and/or costs	“…but don′t feel ready for a full commitment of taking on a pet dog and the responsibilities and costs that that entails.”	
		Flexibility	“I like the flexibility of being able to have a dog when I want…”	
	Benefits to health	Physical health	“I needed to lose weight and could see daily dog walks could only be a benefit to me.”	6 (22.2%)
		Mental health/social connectedness	“I also do this for my mental health.”	
	Interest in dog training/puppy raising		“I used to be a teacher and have been fascinated by the whole process of training these wonderful pups.”	3 (11.1%)
Dog related positive affect-based motivation	Love of dogs/animals		“My partner and I have always wanted a dog of our own since we moved to Melbourne. We are huge dog lovers.”	6 (22.2%)
Dog related positive affect-based motivation	Positive emotion, experience or interaction		“To enjoy having a puppy…”	3 (11.1%)

Percentages were calculated by dividing the number of participants who endorsed a theme by the total number of participants. Individual participants were only able to endorse a theme once but could endorse more than one theme. Hence, percentages in tables do not sum to 100%. Hence, percentages in tables do not sum to 100%.

**Table 9:** CDO motivations for becoming a companion dog owner (*n* = 77).

Meta-Theme	Theme	Sub-Theme	Example Quote	Frequency, *n* (%)
Prosocial based motivation	For own child/family		“To teach son about empathy and caring and to get him to consider another life other than his own.”	18 (23.4%)
	Dog rescue		“I enjoy the challenge and rescuing them.”	15 (19.5%)
Self-related motivation	Benefits to health	Physical health	“Having a dog keeps you active…”	12 (15.6%)
		Mental health/social connectedness	“Socially meeting other dog owners.”	
Dog related positive affect-based motivation	Love of dogs/animals		“Because I love dogs and the house always feels empty if we do not have a dog.”	26 (33.8%)
	Companionship		“Dogs make lovely companions and I like her to accompany me where possible.”	22 (28.6%)
	Positive emotion, experience or interaction		“They make me feel happy and I love having them around.”	12 (15.6%)
	Fond memory of previous dog		“Have always had or had access to a dog. I love their honesty, devotion, unconditional loving nature.”	12 (15.6%)
	Improve life		“I like walking and a dog adds to my life in many ways…”	7 (9.1%)
	Dog chose them		“My dogs chose to live with me, they adopted me”	4 (5.2%)

Percentages were calculated by dividing the number of participants who endorsed a theme by the total number of participants. Individual participants were only able to endorse a theme once but could endorse more than one theme. Hence, percentages in tables do not sum to 100%. One participant did not provide a response to this question. Three responses relating to lifestyle and 10 unique responses are not included in the table.

**Table 10:** SED and CDO meta theme endorsement for feelings towards their dogs as compared to significant others.

Meta-Theme	SED Carer		CDO	
	Number of Endorsements	(%)	Number of Endorsements	(%)
Not comparable to significant other, role-related	18	42.9	6	9.2
Comparable to significant others or more	13	30.9	38	58.5
Comparable somewhat to significant others but less	11	26.2	21	32.3

Percentages were calculated by dividing the number of endorsements for each individual meta-theme by the total number of endorsements made across all meta-themes. Individual participants were only able to endorse a theme once but were able to endorse more than one theme within the same meta-theme.

**Table 11:** SED carer descriptions of their feelings towards SED dog compared towards significant others (*n* = 27).

Meta-Theme	Theme	Sub-Theme	Example Quote	Frequency, *n* (%)
Not comparable to significant others, role-related	Performance/long term success orientated		‘We feel responsible to our puppies success, as we would the success of our children when younger.’	8 (29.6%)
	Responsible for success/nurture/Upbringing		“I see the pups as fantastic companions, and I enjoy the challenge of raising them to be very successful.”	5 (18.5%)
	Level of detachment	Only a carer	“However, we accept that ours is only a carer role, and that our puppy will eventually be leaving us.”	5 (18.5%)
		Easily replaceable	“I love [SED dog] and I love having him around but also looking forward to getting to know a new puppy when he is ready to move on with his training…”	
		Just a dog	“Our puppy is (just) a dog, an intelligent animal that will be leaving us hopefully to bring happiness and more importantly independence to another person. It does not compare to others in our life…”	
Comparable to significant others or more	Same as another pet/dog		“I love my SED puppy very much. “…it doesn’t mean she gets treated any differently to my pet dog. At home, she gets the same love and attention as my dog…”	7 (25.9%)
	Same as family		“He is important for me as normal member of my family.”	6 (22.2%)
Different to significant others, or comparable but less	Different to family/friends.		“It is a dog not a person, so I love them but not in the same way as significant others...”	6 (22.2%)
	Less than family		“I am single, and my children are adults now, obviously I love my children very much and... I love them but family comes first!”	5 (18.5%)

Percentages were calculated by dividing the number of participants who endorsed a theme by the total number of participants. Individual participants were only able to endorse a theme once but could endorse more than one theme. Hence, percentages in tables do not sum to 100%.

**Table 12:** CDO descriptions of their feelings towards dog compared towards significant others (*n* = 77).

Meta-Theme	Theme	Example Quote	Frequency, *n* (%)
Not comparable to significant others, role related	Responsible for success/nurturing/upbringing	“My feelings towards my dog are very deep as there is responsibility to provide everything from a safe environment, food and love.”	6 (7.69%)
Comparable to significant others or more	Same as family	“We loved our 2 dogs very much they are a big part of our family.”	19 (24.7%)
	Prefer to some humans	“…I would say I love my pet more than a lot of humans.”	12 (15.6%)
	More than family	“He is by top priority and my longest valuable relationship in my life so far. He is more intelligent, intuitive and fun to be with then most people I know.”	7 (9.1%)
Different to significant others, or comparable but less	Different to family/friends.	“I love my dog. My feelings towards him are different than those I have for significant others.”	11 (14.3%)
	Less than family	“I love my dog. But my family and kids will always come first.”	10 (12.99%)

Percentages were calculated by dividing the number of participants who endorsed a theme by the total number of participants. Individual participants were only able to endorse a theme once but could endorse more than one theme. Hence, percentages in tables do not sum to 100%. One participant did not provide a response to this question. Ten responses related to health benefits and three unique responses are not included in the table.
